# Self-Assembling Peptides: From Design to Biomedical Applications

**DOI:** 10.3390/ijms222312662

**Published:** 2021-11-23

**Authors:** Sara La Manna, Concetta Di Natale, Valentina Onesto, Daniela Marasco

**Affiliations:** 1 Department of Pharmacy, University of Naples “Federico II”, 80131 Naples, Italy; daniela.marasco@unina.it; 2 Istituto Italiano di Tecnologia, IIT@CRIB, Largo Barsanti e Matteucci, 53, 80125 Napoli, Italy; 3 Centro di Ricerca Interdipartimentale sui Biomateriali CRIB, Università di Napoli Federico II, Piazzale Tecchio, 80, 80125 Napoli, Italy; 4 Institute of Nanotechnology, Consiglio Nazionale delle Ricerche, CNR NANOTEC, via Monteroni, c/o Campus Ecotekne, 73100 Lecce, Italy; valentina.onesto@nanotec.cnr.it

**Keywords:** SAPs, self-assembly, drug delivery, tissue regeneration, biomaterials, hydrogel

## Abstract

Self-assembling peptides could be considered a novel class of agents able to harvest an array of micro/nanostructures that are highly attractive in the biomedical field. By modifying their amino acid composition, it is possible to mime several biological functions; when assembled in micro/nanostructures, they can be used for a variety of purposes such as tissue regeneration and engineering or drug delivery to improve drug release and/or stability and to reduce side effects. Other significant advantages of self-assembled peptides involve their biocompatibility and their ability to efficiently target molecular recognition sites. Due to their intrinsic characteristics, self-assembled peptide micro/nanostructures are capable to load both hydrophobic and hydrophilic drugs, and they are suitable to achieve a triggered drug delivery at disease sites by inserting in their structure’s stimuli-responsive moieties. The focus of this review was to summarize the most recent and significant studies on self-assembled peptides with an emphasis on their application in the biomedical field.

## 1. Introduction

Molecular self-assembly is a natural process driven by various non-covalent interactions, such as electrostatic and/or hydrophobic, aromatic stacking, hydrogen bonding, or metal coordination interactions [1]. The elements of a self-assembling process are macromolecules or their fragments that, under physiological conditions, interact with each another. These elements may be equal or different, and their interaction starts from a disordered state leading to a final ordered form (e.g., well-defined crystals or structured macromolecules) [2,3].

In nature, the self-assembly is a common process essential to the life activities of cells; for example, the formation of cytoskeletons requires the self-assembly of actins and tubulins or the formation of double-stranded DNA molecules [4,5]. Errors in the self-assembly processes can cause serious pathologies, such as neurodegenerative diseases resulting from the abnormal fibrous assemblies of proteins [3,6,7].

Considering these physiological roles, in recent years, the self-assembling process has been largely studied; in particular, self-assembling peptides (SAPs) are arousing great attention as novel biomaterials in different fields such as nanomedicine, nanomaterials, and nanobiotechnology [8,9,10,11]. Generally, SAPs consist of monomers of short (8–16-mer peptides) or repeated sequences able to assemble to form well-ordered nanostructures.

It is well known that peptide-based structures, with respect to synthetic small molecules, possess several advantages like high chemical and biological diversity, higher potency and selectivity for their targets, low toxicity and good membrane penetration, and they are used in different fields [12,13]. Unfortunately, almost all peptides show poor metabolic stability and rapid clearance that renders them not always suitable as drugs [14].

Thanks to their characteristics, SAPs show unique biochemical and physicochemical activities that can overcome the disadvantages of common peptides. Indeed, the modification of the SAPs’ chemical structure allows to regulate their size and morphology (e.g., micro/nanospheres, micro/nanoparticles, etc.) [15,16]. By modifying SAPs’ composition, in terms of length of sequence and amino acidic composition, it is possible to generate SAPs able to form structures with greater selectivity and stability than traditional non-biological materials under extreme conditions of temperature and pH. To reduce unwanted side effects, it is also possible to design peptides able to self-assemble into a particular biological target: the “enzyme-instructed self-assembly of peptides” is a novel strategy used to in-situ trigger the self-assembling process thanks to the action of a specific enzyme [17].

Furthermore, when assembled, SAPs can be used as scaffolds for cell and tissue regeneration [18] and carriers for drug delivery with controlled release, a low ratio of drug loss, a high stability and selectivity, and poor side effects [17,19,20].

Currently, SAPs are also used as a three-dimensional cell culture [21]and as biosensors, such as the Fmoc-FF hydrogel-based biosensor [22]. They are also used in different vaccine formulations, such as Q11 peptide for the intravaginal vaccination [23], and some of them have already reached the pharmaceutical market [24,25,26].

In this review, we describe how to design SAPs using different building blocks and their potential application as drug delivery systems. Furthermore, we summarize the most recent and significant studies on SAPs with an emphasis on their application as drugs in the clinical and biomedical field.

## 2. Self-Assembly Process

Normally, to design functional peptide assemblies, the first step involves the morphological control; indeed, it is important to understand the nature of peptide folding that drives the self-assembly process and creates a range of diverse structures. Depending on the nature of building blocks, it is possible to synthesize SAPs with distinctive properties that are largely used in the biomedical area. The building blocks can be categorized according to their distinctive constituent amino acids and/or to the various bound chains (e.g., polyethylene glycol (PEG) units).

**Figure 1:**
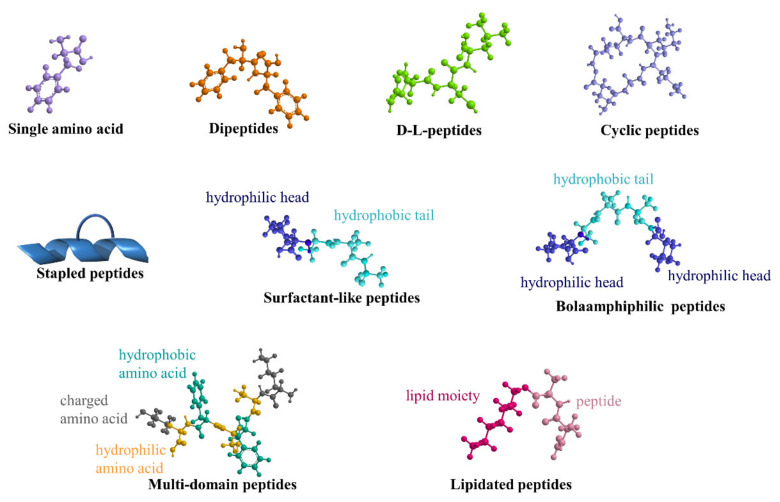
Schematic representation of the most common building blocks to obtain SAPs described in this review.

Herein, the most-used building blocks, intended as peptide monomers able to auto-assemble to obtain SAPs with different morphologies, are schematically reported in Figure 1.

### 2.1. Single Amino Acids

Single amino acids can provide similar advantages of peptides but with the additional advantage of lower costs of production [27]. A first example of a single protected amino-acid-based hydrogel was reported some years ago by Gortner and Hoffman and was explored by Menger et al. These authors showed the hydrogen bonding and π–π interactions of a dibenzoyl cysteine can tune its rheological properties. Another amino acid used as hydrogelator was the cinnamoyl protected phenylalanine. It demonstrated an efficient hydrogelator (its critical gelation concentration (CGC) was between 0.1–2 wt%): this mechanism was pH-dependent and thermally reversible [27]. Xu et al. showed the hydrogelation of a mixture of Fmoc–lysine and Fmoc–valine together with external stimuli-responsive Fmoc–amino-acids able to detect enzymatic processes in bio-analytical applications [28]. The hydrogelation mechanisms of Fmoc–phe was also demonstrated by other groups, and it occurred through pH triggers from basic to neutral/acidic values. Nillson et al. reported on Fmoc–phe, Fmoc–tyr, and a pentafluorinated analogue of Fmoc–phe [29]. In this last one, the presence of fluorine significantly decreased the electron density of the phenyl-ring-tuning hydrogelation mechanism. The role of halogenation was analyzed also through single halogen substitution at ortho or para positions of the benzyl group of Fmoc–phe: the meta substitution led to a fast gelation (3–5 min), the para to immediate hydrogelation (30 s), while the ortho led to a moderate one (30–40 min). These observations were probably due to a perturbation of the π–π interactions [30]. Other studies analyzed the role of the C-terminus of halogenated Fmoc–phe, showing that it is also important in hydrogel-formation-tuning self-assembly behavior. Gelation mechanisms of these derivatives were investigated in comparison to the relative carboxylic acid compounds at neutral or acidic pH. The studies underlined that the hydrogelation of the COOH termini were highly sensitive to acid pH (3.5), while, at pH 7, hydrogel rigidity was significantly decreased [31]. On the contrary, amide derivatives assembled much more rapidly than carboxylic acids at both acidic and neutral pH, but the resultant hydrogels were unstable [31]. These features can be used to tune the bulk properties of hydrogels, providing systems that can be used in different biomedical fields.

### 2.2. Dipeptides

Dipeptides represent the simplest building blocks possible. The most common dipeptide, NH_2_-FF-COOH (F, phenylalanine), derives from the central hydrophobic cluster of the amyloidogenic polypeptide Aβ-42, and it is considered as the smallest homo-aromatic peptide able to self-assemble thanks to intramolecular aromatic interactions. It is able to form highly ordered nanostructures, such as nanotubes, nanowires, nanocrystals, and nanoparticles [32,33]. Fmoc–FF is applied in various fields because of the advantage to be simply synthesized either using solution- or solid-phase peptide synthesis methods trough the Fmoc group as an amine-protecting group [34]. On the other side, Fmoc is base-sensitive, and this entails a problem in the formation of the gelation process, which usually starts at a pH around 10 [35]. With the aim to overpass this limitation, a number of different capping groups were used as a naphthalene-based capping group, which is not base-labile and which brags several sites for additional functionalization, including the carboxybenzyl and cinnamoyl groups, which require hydrophobic peptide sequences such as diphenylalanine to form hydrogels [32,36] or the photoresponsive spiropyran, azobenzene, and dansyl groups [34,37,38].

The use of different N-terminal capping groups is useful for tuning peptide self-assembly and its resultant hierarchical structures. For example, several non-charged FF analogues (e.g., Boc–FF–COOH) are able to generate tubular nanostructures [39], while the conjugation with aromatic groups (fmoc and fluorenylmethoxycarbonyl) then induce the formation of hydrogels thanks to π–π stacking and hydrogen bonding [40]. By introducing a thiol group in the FF peptide, it is possible to regulate the formation of ordered spherical nanostructures. The modification of phenyl side-chains with halogen atoms or nitro substitutions (e.g., di-para-fluoro-F, di-para-iodo-F, and di-para-nitro-F) and/or the addition of phenyl groups (di-4-phenyl-F) strengthens the formation of well-ordered nanostructures [41].

Hydrophobic dipeptides such as LF, LI, IV, VI, and VA (L, leucine; I, isoleucine; V, valine; A, alanine) are also able to generate nanoporous structures, and the pore size can be easily regulated by modifying side chain substituents [42].

### 2.3. D/L-Peptides

Unlike L-peptides that normally are easily degraded by proteases, peptides made of D-amino acids are weak substrates of enzymes. This characteristic made it possible to use D-peptides as excellent carriers to control the release rate of a specific drug, to protect easily degradable active molecules, and to increase their half-life. The insertion of D-amino acids is reported to twist heterochiral self-assembled peptides and renders them able to self-assemble into functional hydrogels [43,44]. Furthermore, the presence of D-amino acids induces faster kinetics and increases the aggregation tendency leading to the formation of more stable and rigid hydrogels [45]. The introduction of even just a single different chirality in a peptide sequence can drastically influence the ability to form supramolecular assemblies: in a recent study, the D-scan of a fragment of nucleophosmin 1 protein (NPM1 268–273) confirmed that the insertion of D-amino acids can modulate and dictate different methods of recognition [11]. Interestingly, a recent study showed that the insertion of both D- and L-amino acids at specific positions along a hydrophobic tripeptide (e.g., L-D-L) allows the formation, under physiological conditions, of hydrogels much more stable with respect to their homochiral analogues that are more prone to precipitation [46].

Self-assembling peptides are widely used in anticancer therapy. Thanks to their higher stability to degradation, hydrogels of D/L-peptides are excellent carriers for anticancer drugs. For example, the peptide Nap-GffyGRGD (Nap: (naphthalen-2-yl)acetic); lower-case letters indicate D-amino acids) has been used as a hydrogelator to connect a tumor-targeting tripeptide (RGD, Arg-Gly-Asp) and to deliver 10-hydroxycamptothecin (HCPT) for the treatment of several tumors (e.g., gastric carcinoma, hepatoma, leukemia, and tumors of the head and neck) [47].

### 2.4. Cyclic Peptides

Cyclic peptides can auto-assemble to form cylindrical structures [48]. These structures are achieved thanks to intermolecular hydrogen bond between each amino acid, forming a β-sheet-like tubular structure. For these characteristics, in comparison to the other peptide self-assembled nanostructures, cyclic peptides preferentially form nanotubes, and the amino acids that compose them are normally arranged with the side chains outside the tube and the peptide backbone inside. Thanks to this chemical structure, the external surface properties and the diameter of the cylinder can be controlled by the suitable choice of amino acid side chains and the peptide lengths. Usually, cyclic self-assembling peptides are obtained by introducing D-amino acids in their peptide sequence: the first self-assembled nanotube was obtained using a sequence of alternating D and L amino acids, cyclo-(L-Gln-D-Ala-L-Glu-D-Ala)_2_ [49].

Interestingly, the cyclization is a novel strategy used to allow cell membrane penetration: for example, self-assembly of cyclic peptide (cyclo[Q-(l-W)4-l] (Q, glutamine; W, tryptophan; l, D-leucine) generates small unnatural transmembrane nanochannels (<1 nm) on the cell membrane for the delivery of anti-cancer drugs such as 5-fluorouracil (5-FU) [50].

### 2.5. Stapled Peptides

Normally, self-assembling short peptides can form ordered assemblies rich in β-sheet structures. However, α-helical structures, the most common secondary structures of natural proteins, are less exploited for the creation of assembled structures. Short peptides containing unmodified amino acids present weak intrinsic helical stability, which hinders their use to generate self-assembled helical structures [51]. One of the main strategies involves the use of helix-promoting noncoded amino acids such as β–amino acids and α-aminoisobutyric acid [52]. Another strategy involves the use of side chain stapling able to enhance the helical tendency [53,54,55,56]. Very recently, a thioether-containing side chain staple was shown to be able to promote the folding of 5-mer peptides into canonical helical structures able to evolve into coiled coil nanofibers [57].

### 2.6. Amphiphiles and Branched Amphiphilic Peptides

This group includes peptides formed of hydrophobic tails (e.g., alkyl chain tails) and hydrophilic peptide head groups. The interaction between these two units could stabilize various supramolecular structures thanks to electrostatic, hydrophobic, and π–π stacking interactions or hydrogen bonds [58,59]. These kinds of peptides could self-assemble into several nanostructures with different morphologies such as nanovesicles, nanotubules, and hydrogels [59,60]. Recently, peptide amphiphiles (PAs) were studied as antitumoral carriers: a series of PAs (Nap-C_12_-VVAAG, Nap-C_12_-VVAAD, and Nap-C_12_-VVAADD) with varying tail groups and C-terminal hydrophilic amino acids have been tested for their capability to self-assemble and form hydrogels to release the antitumoral drug doxorubicin (DOX) [61].

### 2.7. Bolaamphiphilic and Surfactant-Like Peptides

Generally, surfactant-like peptides are constituted by a hydrophilic head linked to a hydrophobic long tail. They tend to self-assemble by forming a polar interface when dispersed in aqueous solution and hiding the hydrophobic tail from water. This behavior allows the formation of nanostructures such as nanotubes or nanovesicles [62]. Most known surfactant-like peptides are the following sequences: Ac-AAAAAAD (A6D), Ac-VVVVVVD(V6D), Ac-VVVVVVDD (V6D2), Ac-AAAAAAK (A6K), Ac-LLLLLLKK (L6K2), and Ac-LLLLLLKD (L6KD) [63]. Depending on the nature of hydrophilic amino acids they could be positively (e.g., lysine, histidine, and arginine) or negatively (e.g., aspartic and glutamic acid) charged.

The difference between bolaamphiphilic and surfactant-like peptides is the number of hydrophilic head groups of the building block: only one head in the surfactant-like peptides, while two hydrophilic groups at both ends separated by hydrophobic spacers in the bolaamphiphiles [64,65].

Given that structural similarity, bolaamphiphiles (also known as bolaform surfactants) show similar properties of surfactant-like peptides. Structurally, they present two hydrophilic extremities and one hydrophobic connection. Interestingly, bolaform surfactants are often used to bind DNA/RNA, and, for this reason, the two heads are usually amino acids with positive charges (KAAAAK (KA4K), KAAAAAAK (KA6K), and RAAAAAAR (RA6R)) able to bind negative residues of nucleotides [66].

### 2.8. Multi-Domain Peptides

Multidomain peptides (MDPs) are a class of self-assembling peptides able to organize themselves in β-sheet motifs, which result in nanofibrous architectures stabilized by a hydrophobic core and hydrogen-bonding networks down the fiber long axis. Generally, all MDPs show the same chemical design, with the core of the peptide constituted by alternative hydrophilic and hydrophobic amino acids and, at the extremities, charged amino acids. By controlling pH conditions, peptide concentration, or salt media composition, it is possible to regulate electrostatic interactions between the peptides and solvent, tuning nanofiber length and hydrogel formation [67]. For example, Lopez-Silva et al. designed a series of peptides creating steric interactions adding neutral hydroxyproline (O) domains and eliminating the need for charged residues to obtain neutral peptide hydrogel and to determine the effect of oligo-hydroxyproline on peptide self-assembly and nanostructure. They showed that O residues give the necessary hydrophilicity thanks to their hydroxyl side chain, and, moreover, they are not able to participate in the N–H backbone hydrogen bond donation necessary for β-sheets development. Furthermore, they inserted the O repeats into peptide sequences to form polyproline type II (PPII) helices, which offer a flexible terminus for the growing of peptide nanofibers by both steric impediment and secondary structure disruption. To study the effects of oligo-hydroxyprolines, MDPs with different number of O residues (O_n_(SL)_6_O_n_, where *n* = 1—6) were investigated: peptide solubility and nanofibers’ length increase with the number of O. Only O_5_(SL)_6_O_5_ was able to form a hydrogel, and this nanostructured hydrogel was able to maintain fibroblast cell viability, while in vivo it was easily degraded over time without promoting a strong inflammatory response [67]. Moreover, the MDP hydrogel can be used to target a specific cellular location or tissue, simply by changing the amino acid sequence generating MDPs sensitive to specific enzymatic degradation or by adding biomimetic sequences derived from cell receptors. In this context, Moore et al. designed MDP hydrogels containing a core with an alternation of hydrophilic and hydrophobic amino acids and charged amino acids at the N- and C- termini to control blood vessel formation. In detail, they added glutamic acid or lysine at the extremities to generate sequences with net negative or positive charges, while as hydrophilic residues, they included glutamine, serine, threonine, and cysteine, and, as hydrophobic residues, they included both aliphatic and aromatic amino acids [68]. Generated peptides resulted endowed with a β-sheet secondary structure bearing hydrophobic residues on one side and hydrophilic residues on the other side, which was shown to be the key for the supramolecular assembly of peptides in nanofibers [69].

### 2.9. Lipidated Self-Assembly Peptides

Lipidated peptides exhibited attractive features [70] because of the possibility to modulate their lipophilicity, thus influencing their absorption, distribution, metabolism, excretion, or bioavailability [71]. Generally, lipidation increases the stability and the half-life of a drug in vivo, facilitating its binding to a carrier protein as serum albumin: for example, the addition of a palmitoyl chain, which delays renal clearance by the kidneys and thus prolongs biological activity of the drug [72]. Hutchinson et al. analyzed the self-assembly behavior of lipidated peptides containing palmitoylated fragments of the gastrointestinal peptide hormone PYY3–36. This peptide belongs to the pancreatic peptide (PP), and it has high selectivity for the Y2 receptor, which is associated with food intake [73,74]. The authors analyzed the self-assembly in aqueous solution of modified PYY3–36 peptide with different lipid moieties as hexadecyl or octyl lipid chains within its α-helical core. The lipidation induced the formation of micellar structures at low pH, while fibrillar structures were obtained at high pH only for the palmitoylated derivatives. The effects of the palmitoyl chain attached at the N-terminus of two N-terminal truncated fragments of PYY3–36, constituted by the first six and eight amino acid residues of the entire sequence, respectively, were investigated. The results revealed that the lipidation of short fragments allowed the formation of large micelles with an association number (p ≈ 80) with respect to the small “micelles” formed by the lipidated long PYY3–36 peptide (p = 5–10). In addition, the two lipopeptides form β-sheet fibrils on drying, bringing the formation of “amyloid-like” structures. In vivo studies indicated that palmitoyl moieties allowed peptides to fuse with the cell membrane, potentially acting as a transducing molecule [73].

These outcomes can be useful in the development of proto-globular protein model systems for the creation of therapeutic agents based on lipidated fragment molecules [73].

## 3. Co-Assembly Process

**Figure 2:**
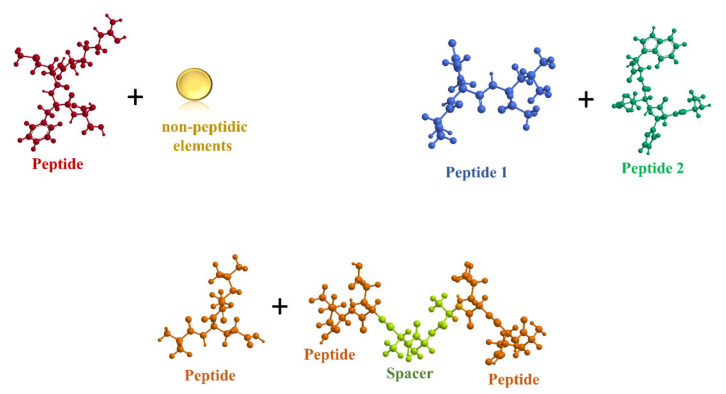
Most common strategy to obtain co-assembly structures.

Another elegant strategy to obtain supramolecular assembled structures consists in the co-assembly process. This is a process in which two different elements can associate to form a single network able to produce defined multifunctional structures with unique architectures and functions (Figure 2) [75].

According to the purpose, several strategies could be used to design co-assembled structures. One approach involves the association of two peptides with the same sequence but one of them bearing typical motifs, such as the EAK16-II peptide that mixed with its histidinylated form, which is used to induce a strong aggregation of epithelial cells and wound closure [76].

It is also possible to mix one peptide with a longer one formed by two sequences of the same peptide and connected by a spacer: for example, the peptide FEFEFKFK linked to its double-length peptide, FEFEFKFK-GG-FKFKFEFE, generates fibers with increased stability and elasticity [77].

Another strategy involves the use of different types of peptides combined with different non-peptidic structural elements [78]. Recently, a multivalent structure composed by the combination of three peptides and carbon nanodots (CNDs) has been described. As previously mentioned, the advantage of this kind of a co-assembled structure is that it shows various combined functions: in this case, the recognition ability of peptides and the important catalytic activity of CNDs. Thanks to these characteristics, it has been applied as a potential biosensor for the detection of transglutaminase 2 (TG2), a multifunctional enzyme linked to several pathologies (e.g., cancer, cardiovascular disease, celiac disease, and neurodegenerative diseases) [79]. In detail, the co-assembled structure is formed by three peptide sequences: (i) P1 (KLSEKEKEKEGGGGSC) that can specifically recognize TG2 and that is anchored on an Au-electrode; (ii) P2 (HQSYVDPWMDHHHYY), a link peptide used to connect P1-TG2 and P3; and (iii) P3 (GHHYYGHHYY), a tyrosine-rich sequence able to spontaneously create a co-assembled structure with CNDs thanks to the π–π stacking. To detect the presence of TG2, the Au-electrode was immersed in a solution containing 3,3′,5,5′-tetramethylbenzidine (TMB) and peroxide. The CNDs’ peroxidase-mimicking activity was measured through an amperometric detector. In the absence of TG2, P1 was not able to link P2, and consequently, P3/CNDs did not assemble and was unable to show peroxidase activity [80].

Interestingly, Ajovalasit A. et al. very recently described an innovative co-assembled structure constituted by a peptide amphiphile and a natural product, the tamarind seed xyloglucan (XG). This natural compound has anti-inflammatory properties and positive effects on skin re-epithelization. Generally, the combination of cationic PAs with polysaccharides can create structures similar to membranes, which are useful for skin applications. In this work, a cationic PA (palmitoyl-VVVAAAHHH) and a carboxylated XG (CXG) were co-assembled through the injection of PA solution drops inside CXG solution. The resulting CXG-PA hydrogel revealed to be able to significantly close wounds in in vivo studies, acting as a potent therapeutic for skin lesions [81].

As demonstrated by these examples, the co-assembly process is a novel and interesting approach to obtain innovative and multifunctional biomaterials.

## 4. Self-Assembly Peptides in Biomedical Applications

### 4.1. In Diagnostics

Magnetic resonance imaging (MRI) is a growing research discipline that allows to image intact and opaque tissues in three dimensions at cellular scale in a non-invasive manner [82]. It is based on the variation of the relaxation times of water molecules modulated by the presence of contrast agents; high concentrations of contrast agents are required to improve the signal-to-noise ratio and the sensitivity of the images, and this increases cytotoxicity [82]. Therefore, self-assembling contrast agents able to increase sensitivity in the analyzed area preserving the surrounding tissues can be conceived. Self-assembling contrast agents are highly efficient because being composed by small molecules, they show longer cellular retention and higher cellular uptake prior to self-assembly [83].

Cao et al. designed a gadolinium (Gd)-based MR contrast agent that is susceptive to furin, a protease that is overexpressed in cancer cells. After intracellular disulfide reduction, the structure condenses into amphiphilic dimers, which subsequently self-assemble into Gd-nanoparticles (NPs). Gd-NPs assembly causes the increase in the local intracellular concentration of Gd with a higher molecular weight with consequent higher relaxivity. In vivo studies on tumor-xenografted mice reported the preferential presence of assembled Gd-NPS in the tumors, which enhanced the contrast of MRI images as compared to non-cleavable Gd-containing peptides [84]. In another study, Gallo et al. developed DTPA(Gd)-PEG8-(FY)3 or DOTA(Gd)-PEG8-(FY)3 peptides forming soft hydrogels modified with gadolinium complexes as MRI contrast agents. (FY)3 moiety was able to self-assemble in water into cross-β nanostructures. The low in vitro cytotoxicity and the high relaxivity value of these nanostructures suggest their potential application as an injectable MRI contrast agent [85].

### 4.2. In Luminescence and Optoelectronics

Fluorescence bioimaging plays an essential role in biomedical research and treatment, being a widely used technique, for example, in cellular studies to visualize individual functional sites, in applied therapeutics to identify tumors, and to track conformation processes in real time [86]. The main challenge consists in delivering fluorescent dyes in natural biological forms with greater light-emitting features and amplified and lasting spontaneous emission. Machnev et al. compared RhoB-PEG1300-F6, a biocompatible peptide derivative material covalently linked to rhodamine B (RhoB), with high concentrated solutions of the same fluorophore and showed that pure RhoB solution demonstrated 25% smaller gain values compared to the dye bound to the peptide derivative material [86].

Interestingly, for applications of light diagnostics and therapy in precision medicine, Apter et al. developed biocompatible visible fluorescence light (FL)-delivering probes. Indeed, PEGylated phenylalanine peptide derivatives (PEG-F6) self-assembled into hybrid nanostructures forming β-sheets that acquire the visible FL property found in β-sheet amyloid fibrils associated with neurodegenerative diseases [87].

Self-assembly peptides have even been used for light-activated mechanical actuation in living systems. These light-responsive materials are composed by peptide amphiphile supramolecular polymers with a crosslinked spiropyran network, which expel water in response to visible light. Shaped films of these materials were able to generate a bending and crawling motion under photo-actuation [88].

### 4.3. For Bioprinting

Three-dimensional (3D) bioprinting enables to design complex architectures including different cell types and microenvironments in a rapid, precise, and reproducible manner. One major challenge in bioprinting technologies is the development of suitable bioinks that maintain the shape of the printed construct and that provide a microenvironment allowing cell survival and physiological functions [89]. Different bioink materials are commercially available for 3D bioprinting, but the majority of them include steps that might damage cells as crosslinking by UV or chemical polymerization to transform pre-polymeric viscous solutions into a stable scaffold. In addition, the high viscous nature of these peptide bioinks implies the requirement of extrusion-based printing, in which shear forces exerted on cells extruded from a nozzle of micron-sized diameter can affect their viability and functionality [90].

Ultrashort peptides composed of three to seven amino acids are attractive candidates as bioinks since they can retain high water content preventing cellular dehydration during the printing process [91,92]. Such peptides self-assemble into nanofibrous hydrogels forming biomimetic extracellular matrices with controllable properties designed at the molecular level. Natural peptides as EAK16, RADA16, and elastin-like and silk-like polypeptides can fold into β-sheets, which subsequently form nanofibers thanks to the periodic repeats of hydrophilic and hydrophobic amino acids [91,93]. RAD16-I-based hydrogel was optimized to build 3D predefined structures by 3D printing by Cofiño et al. Indeed, RAD16-I was unable to maintain the designed pattern of 3D printed constructs because of the low viscosity of the SAP, and bioink viscosity was increased by adding methylcellulose (MC). The resultant pattern displayed high shape fidelity and stability [94].

Alternatively, self-assembling can be derived from ABA multi-domain short peptides (the B domain contains hydrophilic and hydrophobic amino acids; the A domain contains charged residues), β-sheet nanotapes, and β-hairpin structures, which subsequently aggregate into fibrils [91]. For example, Jian et al. employed two 9-fluorenylmethoxycarbonyl-dipeptides oppositely charged on their terminal residuesas bioinks in a droplet-based 3D bioprinting. In situ gelation during layer-by-layer deposition was achieved by electrostatic interactions between oppositely charged layers without additional cross-linking procedures. The hydrogels had tunable mechanical properties (Young’s modulus from 4 to 62 kPa) and controllable biodegradability (from days to weeks) [95]. Rauf et al. developed two self-assembling tetrameric peptides (Ac-Ile-Val-Cha-Lys-NH_2_ (IVZK) and Ac-Ile-Val-Phe-Lys-NH_2_ (IVFK)) forming hydrogels during 3D bioprinting utilizing physiological buffers at body temperature [91].

### 4.4. As Antifouling, Antimicrobial, and Antiviral Agents

Biofouling is the unwanted accumulation of microorganisms such as fungi, algae, bacteria, sponges, and barnacles on surfaces. It represents a great issue in many sectors such as food, water, marine, and biomedical industries. For example, undesired accumulation of bacteria on biomedical devices causes infections [96]. To resist biofouling, surfaces can be coated with (i) an antifouling film that is a bioactive or a chemically active material that prevents microorganism adhesion to the surface [97] or (ii) an antimicrobial coating that resists biofouling by killing the organisms [98]. Coatings comprise antibiotics, biocidal agents, nanoparticles, polycationic materials, and polymers. Unfortunately, these solutions suffer from drawbacks such as the absence of long-term stability, great toxicity, and scarce biocompatibility [98]. In this context, peptide-based materials thanks to their biocompatibility, low toxicity, and biodegradability have gained considerable attention as antifouling agents.

For example, Maity et al. designed a self-assembling tripeptide into an antifouling coating on different materials such as metals, metal oxides, and polymers. The peptide was formed of three elements: (i) an adhesion unit (the amino acid 3,4-dihydroxyphenylalanine), (ii) a self-assembly unit (two phenylalanine residues that directed self-assembly through π–π stacking), and iii) an antifouling unit (phenylalanine residues were fluorinated forming a hydrophobic-fluorinated material). Such hydrophobic coating prevented undesirable adhesion of proteins and bacteria, revealing over a 90% reduction in their adhesion compared to uncoated surfaces [99].

Even ultrasmall gold nanodots (Au NDs, size ≈2.5 nm) immobilized with surfactin (SFT) and 1-dodecanethiol (DT) have been used as highly efficient antimicrobial agents. SFT is a cyclic lipopeptide with a sequence of Glu-Leu-D-Leu-Val-Asp-D-Leu-Leu linked to a C11–15 β-hydroxy fatty acid. SFT/DT-Au NDs were obtained self-assembling SFT on DT-anchored Au NDs trough the hydrophobic interactions between the alkyl chains of DT and SFT. Hybrid gold nanodots exhibited very high antimicrobial activity, including to multidrug-resistant bacteria, thanks to the synergistic effect of SFT and DT–Au NDs in penetrating and disrupting the bacterial membrane [100].

Apart for antifouling activities, SAP assemblies can be employed to design viral mimetics as they can act both as structural components and as functional domains that promote cell entry, selective binding, or a specific activity such as catalytic or antimicrobial activities [101]. SAP-based viral mimetic designs can be divided into two categories. First, there are capsid reconstruction strategies according to which structural units of the peptides contribute to the final supramolecular morphology. For example, capsid morphology has been reconstructed from nanospheres self-assembled from β-annulus peptide segments of Tomato bushy stunt virus and Sesbania mosaic virus [102]. The second category includes simplified virus-like complexes in which functional peptide units are conjugated with RNA or DNA fragments [101]. Cao et al. designed I3V3A3G3K3 peptide to induce DNA condensation into virus-mimicking structures. First, the peptide bound to the DNA chain through electrostatic interactions and then self-associated into β-sheets through hydrophobic interactions and hydrogen bonding. In this form, the peptide assisted DNA packaging, mimicking the nature of the virus capsid [103].

### 4.5. Self-Assembling Peptides as Drug Delivery Systems

Over the last decades, many advances have been made in the use of SAPs as nanomaterials for drug delivery. Ideally, drug delivery systems have to be biocompatible and must protect drugs by avoiding degradation while maintaining their stability and activity [104,105,106,107,108]. In addition, such systems should ensure a controlled and prolonged drug release to a specific targeting site, without toxic effects to the other cells, and to be finally freely eliminated from the body [109,110].

Because SAPs are extensively present in living organisms (e.g., the double-stranded DNA molecule or the tobacco mosaic virus (TMV), which auto-assembles into rod-like nanostructures), they possess natural biocompatibility and biodegradability [111,112] and enhanced drug delivery efficiency since they can improve cellular uptake by offering specific ligands that bind on the target cell receptors [111,113].

**Figure 3:**
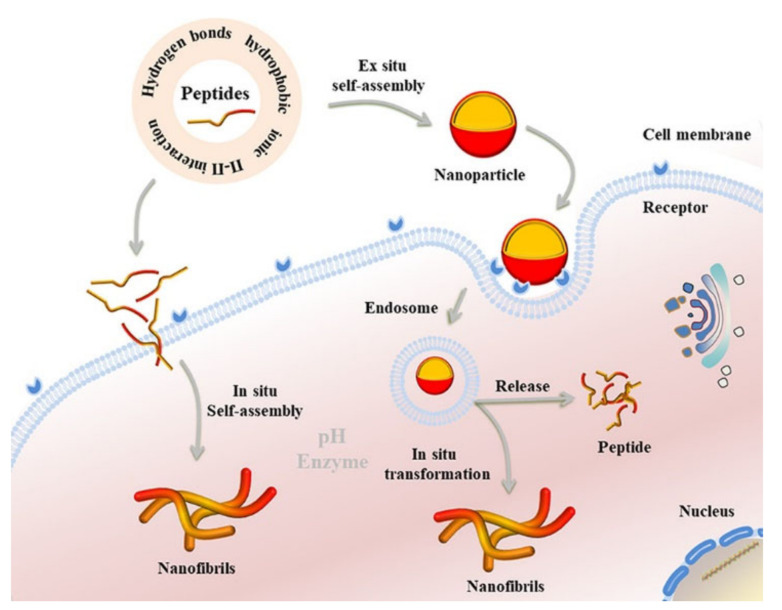
Peptides self-assemble thanks to noncovalent interactions, which mainly include hydrogen bonds, hydrophobic, ionic, and π−π interactions. Self-assembly nanomaterials can be designed to be specifically constructed ex situ, in situ, or to underlie an in situ morphological transformation. Figure reproduced from [109].

SAP-based nanomaterials for drug delivery find most of the applications in cancer therapy, and their design strategies can be divided in three categories: ex situ self-assembly, in situ morphological transformation, and in situ self-assembly of peptides [109] (Figure 3).

In ex situ construction, SAP nanomaterials can be produced in many structures (i.e., nanofibers [114], nanoparticles [115], hydrogels [116,117]), which are internalized within cells by endocytosis. In particular, delivery process includes several phases: blood circulation and accumulation, cell penetration, cell internalization, and finally drug release [109].

The advantage of ex situ SAPs is that SAP nanomaterials can carry drugs within tissues and cells preventing unspecific cellular uptake and clearance. In addition, they can be designed to degrade upon local cues, inducing the drug release. Drug release can be triggered at a specific location by responsive factors such as enzymes, pH, and reducing conditions.

Drug molecules are in general encapsulated physically or by chemical conjugation, and they can be released by the disassembling of nanostructures or the cleavage of conjugated bonds. For example, Ding et al. physically encapsulated 10-hydroxycamptothecin (HCPT) in hydrophobic regions of self-assembling peptide nanostructures and demonstrated sustained drug release, and therefore better in vivo antitumor activity, with respect to the non-encapsulated HCPT [47]. To carry pirfenidone, Ji et al. developed a matrix metalloproteinase-2 (MMP-2)-responsive peptide-hybrid liposome (MRPL). Since an acidic microenvironment is a cancer hallmark, the pH-responsive structure could specifically release pirfenidone at the pancreatic tumor site. MMP-2 was overexpressed, and the secrete extracellular matrix was down-regulated enhancing the efficiency of penetration of the drug into the tumor. The pH response was even exploited by Stupp et al. to encapsulate camptothecin, a hydrophobic drug used for chemotherapy, by fabricated nanofibers with histidine peptide amphiphiles. Due to the low pH of the tumor environment, histidine residues were protonated, producing electrostatic repulsion; therefore, nanofibers disassembled, and camptothecin was released [118].

To use the reducing conditions in the tumor as the responsive element, Yang et al. developed a hydrogel formed by a short peptide derivative Nap-GFFYGD. At the C-terminal of the peptide, an H_2_O_2_-responsive thiazolidinone was carried out. When in contact with the tumor microenvironment, thiazolidinones were detached from H_2_O_2_, activating the gel−sol phase transition of the hydrogel and the release of gemcitabine [119].

As mentioned above, drugs can be chemically encapsulated within SAP nanomaterials by covalently linking drug molecules with the peptide. To release drugs to the target drug, peptide links can be cleaved by either natural cleavage or specific cleavage when responsive linkers are introduced. For example, Ding et al. designed a supramolecular nanosphere conjugated with cell-penetrating peptides and Taxol. The nanospheres continuously released Taxol thanks to the bond hydrolysis process [120].

To develop enzyme-responsive systems, it is possible to introduce linkers between peptides and drugs that are cleaved by enzymes expressed only in cancer cells. Tung et al. developed in aqueous solution an SAP gel comprising three portions: (i) a urokinase plasminogen activator (uPA) protease-sensitive motif, (ii) a β-sheet peptide, and (iii) a therapeutic peptide. When uPA was present, the gel became damaged, and the therapeutic peptides were released [121]. Delehanty et al. employed quantum dot-based nanoparticles assembled with a cell-penetrating peptide and Dox-conjugated to an assembly peptide by diverse linkages such as hydrazine, disulfide, and ester. These linkages were cleaved by acidic pH, highly reductive cytosolic glutathione, and esterases, respectively, inducing Dox release [122].

Numerous works based on ex situ construction of SAP nanomaterials are reported in the literature [114,119,120]. However, several issues still need to be addressed. Surface properties are difficult to be controlled; in particular, during the cell internalization phase, nanoparticles (NPs) can underlie either lysosome capture and degradation, with consequent drug inactivation or expulsion via exocytosis, leading to low accumulation of NPs and drugs in cancer cells. For this reason, researchers developed in situ transformable SAP systems to increase the accumulation and preservation of drugs in tumors.

Smart self-assembled peptides showed self-transition properties as morphology transformation was either spontaneous or under stimuli (pH, light, and enzyme) [123]. Morphology’s role during drug transport is pivotal. Indeed, cellular uptake of NPs is faster than nanofibers; additionally, NPs’ circulation time is ten times shorter than nanofibers [124]; conversely, nanofibers show prolonged release and encapsulation efficiency compared with NPs [118,125]. For example, Gianneschi et al. developed NPs conjugated with matrix metalloproteinase (MMP)-cleavage peptide sequences. These NPs were enzyme-responsive, and when they arrive at the myocardial infarction target, they underlie a morphological transformation in nanofibers, with consequent longer-term retention and accumulation at the infarction site [125]. In a later work, the authors used the same NPs as carriers to deliver paclitaxel, a chemotherapy drug. When exposed to MMPs, peptides were cleaved, and NPs experienced both morphological and dimensional transformation from nanometric spherical micelles to micrometric assemblies, with consequent accumulation and retention of drugs at the cancer site [123].

Another responsive factor inducing morphological transformation of nanomaterials are endogenous reactive oxygen species (ROS) around mitochondria present in most cancer cells. In this regard, Cheng et al. developed polymer–peptide conjugates formed by three sections: (i) a β-sheet-forming peptide KLVFF conjugated with poly(ethylene glycol), (ii) a mitochondria-targeting cytotoxic peptide KLAK, and (iii) a poly(vinyl alcohol) backbone. The self-assembled NPs could be internalized by cells and target mitochondria. Since ROS were overproduced around mitochondria in the tumor cells, the thioketal linker was cleaved, leading to transformation from NPs to nanofibers, exposing KLAK that caused selective cytotoxicity against cancer cells [126].

Dynamical morphological transformation is an effective strategy to enhance drug accumulation and retention in target sites. However, most of the nanomaterials passively target the tumor, and active targeting of cancer cells by targeting ligands on the material still cannot afford challenges such as tumor heterogeneity and endosomal escape [127]. Consequently, only less than 1% of the nanomaterials actually accumulate at the target site [128], being mostly phagocytized or accumulated in the liver and spleen, resulting in inefficient drug delivery [129]. Therefore, an alternative approach was developed by researchers, in situ self-assembly, according to which small molecules can self-assemble in situ upon specific stimuli.

Compared to ex-situ materials, the in-situ SAPs in the monomolecular state have several advantages: (i) small molecules circulate in the blood with high penetration in the tumor sites; (ii) rapidly clearing from liver and kidneys with consequent decreased systemic toxicity; (iii) precise and efficient retention and accumulation in the targeted regions thanks to their self-assembly into SAP nanomaterials, bringing a high drug delivery efficiency.

Self-assembling peptides for drug delivery can be conjugated with drugs as chemotherapeutics, fluorophores, or cytotoxic peptides to realize peptide−drug or peptide-toxic conjugates. Usually, to realize drug delivery to targeting regions, drugs are covalently linked to the precursor molecules, and peptides self-assemble in situ [109]. Once monomeric peptides enter in the physiological environment, different trigger factors can be used to drive their self-assembly such as low pH of tumors, upregulation of specific enzyme expression, or photochemical reactions.

For example, pH-responsive groups (cis-aconitic anhydride (CAA)) were conjugated to cytotoxic peptide containing the functional module (KLAK) by Cong et al. These groups were able to self-assemble in situ at acidic pH. Under physiological pH conditions, the carboxyl groups of CAA were exposed, enhancing the hydrophilicity of the molecules. In the tumor microenvironment, CAA was hydrolyzed, increasing the hydrophobicity of the molecules and inducing their self-assembly into NPs. NPs were internalized by tumor cells, and KLAK restored the α-helix structure due to a low pH, with cytotoxic effects [130]. Alternatively, to design enzyme-responsive peptide precursors, Feng et al. utilized carboxylesterase and alkaline phosphatase. When alkaline phosphatase dephosphorylated these peptide precursors, they self-assembled; conversely, nanofibers disassemble by carboxylesterase-induced cleavage of the ester bond on the small molecules [131]. Another important factor is the light. Indeed, it could be employed as a trigger factor to induce self-assembly of peptide precursors integrating photothermal and thermal-response molecules within the peptide. In particular, the cytotoxic peptide CGGG (KLAKLAK)_2_, was conjugated with the thermoresponsive polymer chain poly(β-thioester) and the photothermal molecule indocyanine green. When irradiated by a near-infrared laser, indocyanine green prompted a temperature increase provoking a collapse of poly(β-thioester) and the disruption of hydrogen bonds between water and thermoresponsive molecules that brought to the hydrophobic shrinkage and in situ self-assembly into nanoparticles at the tumor sites. Subsequently, cytotoxic peptides were internalized into tumor cells, and their α-helical structures damaged the mitochondria, inducing cancer cell apoptosis [132].

The in situ assembled nanomaterials can reach tumor regions in monomeric state, be accumulated, and can be rapidly expulsed by the liver and kidney. Moreover, compared to the other strategies discussed above, they have faster release kinetics, improved mitochondrial disruption, and greater cytotoxicity [132].

However, even if SAP nanomaterials show several advantages in drug delivery, some issues need to be solved to translate research to clinics. There are just few complete studies on the pharmacokinetics of SAP materials [130,131].

### 4.6. Self-Assembly Peptides for Tissue Engineering and Regenerative Medicine

Self-assembly peptides represent the ideal building blocks to produce scaffolds providing supportive 3D environment for cell adhesion, migration, differentiation, and proliferation [133]. To be suitable for tissue engineering, SAP structures need to imitate original tissue biomechanics, permit interaction with cytokines involved in tissue regeneration by carrying multiple functional motifs, and possess an absorption time comparable with tissue regeneration kinetics, geometry, and porosity facilitating new tissue invasion and cytokines diffusion [8].

Currently, SAP have been widely employed in hard-tissue regeneration to emulate the biomineralization of bone. Freeman et al. used peptide amphiphiles including a hydrophobic alkyl tail attached to (i) a charged sequence of amino acids with a strong β-sheet tendency, (ii) cell-adhesive RGD ligands, and (iii) a phosphorylated serine residue that attracts calcium ions, promoting the mineralization of bone minerals such as hydroxyapatite [Ca_10_(PO_4_)_6_(OH)_2_] [134].

Negatively charged-amphiphilic SAPs based on three glutamic acid (E) units represent ideal templates for the deposition of hydroxyapatite crystals creating a bioactive nanofibrous scaffold, which induced calcium phosphate mineralization and consequently enhanced osteogenic differentiation of osteoblast-like cells [135].

**Figure 4:**
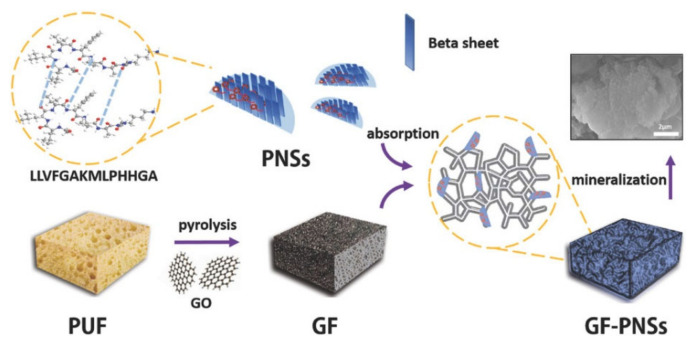
Schematic representation of peptide self-assembly into 2D nanosheets and biomimetic fabrication of 3D graphene foam–peptide nanosheets–hydroxyapatite minerals. Figure reproduced from [136].

Functional peptide sequences were used to create 3D graphene foam (GF)-based hydroxyapatite minerals. GF foam was prepared by coating polyurethane foam (PUF) template in graphene oxide (GO) solution, followed by PUF removal by pyrolysis. The peptide molecule LLVFGAKMLPHHGA was first self-assembled forming 2D peptide nanosheets (PNSs) that were then noncovalently conjugated onto the graphene foam (Figure 4). The obtained scaffold exhibited an adaptable shape, a low weight, and high porosity, providing potential applications for drug delivery and bone tissue engineering [136].

To provide sufficient bioactivity meeting the requirements of clinical applications, Quan et al. developed 3D self-assembled hydrogel scaffolds based on bone morphogenetic protein-2 biomimetic peptide (BMPBP), which is a powerful osteoinductive cytokine. These supports significantly accelerated the in vivo bone regeneration in rat cranial bone defects [137].

In the field of neural tissue engineering, Motamed et al. developed self-assembly peptides composed of β-amino acid building blocks, which, when N-acetylated, generated a network of nanofibers in aqueous solution, forming mechanically stable hydrogel at physiological conditions. Such hydrogels had similar stiffness to brain tissue and promoted neural cell adhesion and proliferation [138].

A self-assembling peptide hydrogel was reported also as a cell carrier for nucleus pulposus of intervertebral discs engineering. The peptide was based on FEFEFKFK (F: phenylalanine, E: glutamic acid, and L: lysine) chains, which possess hydrophilic and hydrophobic faces. Therefore, when solution ionic strength and pH increased, the chains self-assembled into anti-parallel β-sheet nanofibers. After 3D culture of nucleus pulposus cells, upregulation of nucleus pulposus-specific genes pointed out successful tissue regeneration with high cell viability [139].

Interestingly, for suturing small vessels, Smith et al. developed a multiphase transitioning peptide hydrogel that could directly be injected into the lumen. The SAP contained a photocaged glutamic acid and formed a solid hydrogel in the syringe based on β-hairpin interactions. Upon application of a shear, peptides unfolded, and hydrogel dissolved and could be delivered to the lumen of the collapsed vessel. Upon removal of the sheer, the hydrogel network quickly recovered. After suturing was complete, intravascular gel was dissolved initiating the gel–sol phase transition by irradiation with light [140].

As demonstrated by these examples, the self-assembly of various types of peptides shows promising applications in tissue engineering.

## 5. SAPs in the Pharmaceutical Market

The translation of SAPs into commercial drugs requires: (1) an improvement of activity over current drugs on the market; (2) lower or similar costs than the competing products; (3) no toxicity for animal and human tissues; and (4) approval by regulatory agencies (European Medicines Agency, EMA; US Food and Drug Admnistration, FDA) for widespread uses [8].

Currently, SAPs are increasingly used in biomedical fields, and many are already under clinical studies [2,141]. Here, we focused our attention on SAPs that already reached the pharmaceutical market, reported in Table 1 highlighting their structural characteristics and their biological advantages.

### 5.1. RADA16: Hemostatic Agent for Surgery

**Figure 5:**
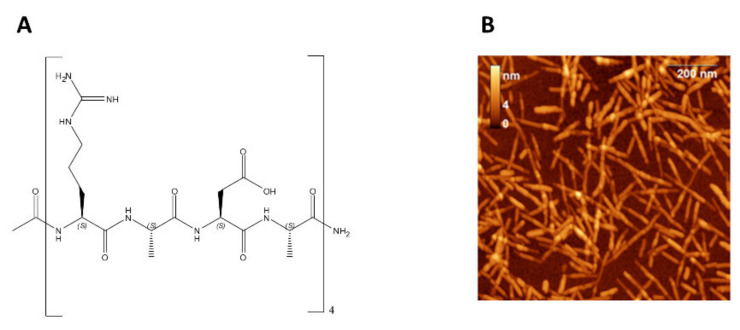
(**A**) Chemical structure of RADA16. (**B**) AFM images of RADA16 fibrils. Figure reproduced from [147].

RADA16 was designed with the intention of mimicking the self-assembly properties of a natural SAP named EAK16. This SAP was unexpectedly discovered in a natural protein as an unusual repetitive fragment (AEAEAKAKAEAEAKAK) [144,145]. To mime this SAP, RADA16 was planned using similar charged natural L-amino acids, in particular: arginine (R) and aspartic acid (D) instead of glutamic acid (E) and lysine (K) and alanine (A) instead of glycine (G) to reduce flexibility of the peptide chain and to guarantee high stability of the self-assembled nanofibers. The obtained structure of RADA16 is reported in Figure 5 [146].

As suggested by the chemical structure, RADA16 is an amphiphilic polypeptide with four repeated homologous sequences (RADA). All free active groups (amine functional group, –NH_2_; carboxylic acid functional group, –COOH) allow the formation of exceptionally stable and highly ordered β-sheet structure through the formation of different types of bonding (e.g., hydrogen, ionic, and hydrophobic bonds) [148]. Following these bonds, in contact with blood or other physiological fluids, a biocompatible and stable hydrogel can form. In particular, it is able to self-assemble into a scaffold that mimics the human extracellular matrix (ECM), and the hydrogel is formed by a network of fibers (diameter 10–20 nm) and pores (diameter of 50–200 nm) that mime the mesh structure of natural collagen (Figure 5B) [147].

Thanks to its properties, RADA16 is currently used as a homeostatic agent for surgery, with the commercial name PuraStat^®^ (prefilled syringe, 2.5% *w*/*v* aqueous solution). It is a CE-marked class III medical device for hemostasis during surgery and for bleeding from small blood vessels. It is also indicated for the reduction in delayed bleeding after gastrointestinal endoscopic submucosal dissection in the colon. The major advantage of using this drug is that it is safe with no side effects or contraindications having emerged. PuraStat^®^ is currently sold in Europe, Australia, New Zealand, Asia, and Latin America [8].

In recent years, many researchers have exploited RADA16 to bind different peptides, obtaining applications in various biomedical fields, such as 3D cell culture, tissue repair, and delivery systems. Interestingly, several sister products are commercialized. PuraSinus^®^ (also 2.5% RADA16) is used intraoperatively to prevent adhesion formation and acts as an adjunct to wound healing after nasal surgery (US Food and Drug Administration, 2021). Another analogue is PuraDerm^®^. This drug is used in the United States as a topical wound dressing for the treatment of superficial or deep wounds like diabetic, decubitus, and leg ulcers or surgical wounds [24].

### 5.2. PF_11_-4: Treatment of Dental Caries

**Figure 6:**
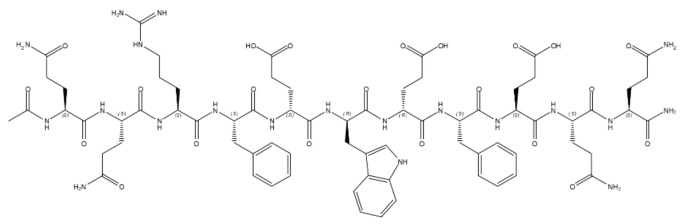
Chemical structure of PF_11_-4.

Another SAP that reached the pharmaceutical market is P_11_-4, which is sold under the registered trademark of Curodont^TM^ Repair. It is a synthetic α-peptide constituted by 11 amino acids (Table 1, Figure 6) containing hydrophilic (glutamine, arginine, and glutamic acid) and aromatic (phenylalanine and tryptophan) side chains. Glutamine residues at both extremities drive the self-assembling process, lead to the formation of β-sheet structures, and further form higher-order nanostructures (e.g., nanofibers and fibrils).

P_11_-4 is used to treat dental caries (remineralization and regression in early dental caries) and in particular to allow enamel regeneration [149,150]. Indeed, when monomeric SAP P_11_-4 is injected in the carious lesion surface, it is able to diffuse into the lesion and self-assemble into nanotapes forming a hydrogel network that mimics the endogenous microenvironment necessary for enamel deposition. This phenomenon is possible because the hydrogel network presents inside its mesh a series of binding sites for calcium ions, which are necessary to form the mineral of the teeth named hydroxyapatite [151]. Moreover, the hydrogel formation is pH sensitive and thus allows control of matrix activity and the place of formation [25,150].

### 5.3. Lanreotide: Treatment of Neuroendocrine Tumors

The third SAP currently on the market is lanreotide acetate. Lanreotide is an analogue of a natural chemical called somatostatin. Somatostatin is endogenously produced by the hypothalamus, and it is normally involved in the regulation of growth hormone as well as inhibiting the release of serotonin, gastrin, secretin, motilin, and pancreatic polypeptide. The principal problem in using it as a drug is its chemical instability: in the body, it is broken down within minutes of its release. To overcome this problem, lancreotide has been developed [152].

**Figure 7:**
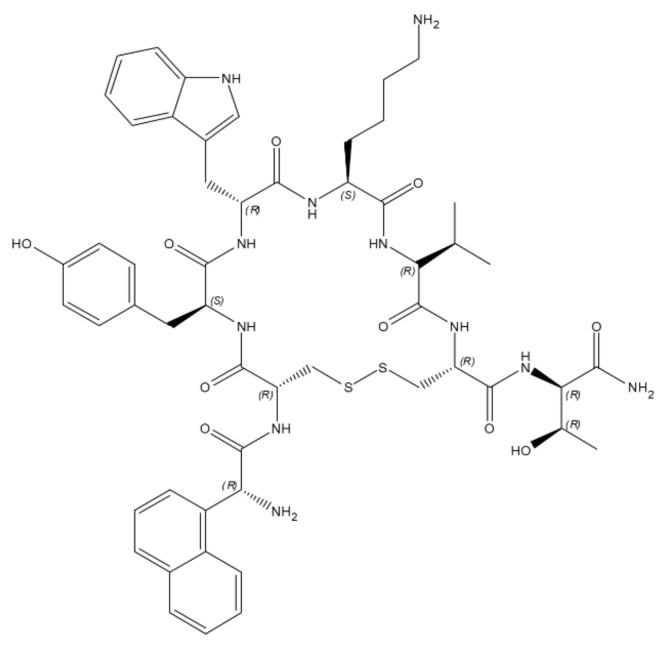
Chemical structure of Lancreotide.

It is chemically known as [cyclo S-S]-3-(2-naphthyl)-D-alanyl-L-cysteinyl-L-tyrosyl-Dtryptophyl-L-lysyl-L-valyl-L-cysteinyl-L-threoninamide, acetate salt], and its structure is reported in Figure 7 [153]. The cyclic structure obtained through the disulfide bridge is stabilized by intramolecular hydrogen bonds.

This cyclic octapeptide in water can form noncovalently bonded dimers. The dimers thanks to noncovalent forces (e.g., hydrogen bonds, hydrophobic interactions, and π–π stacking of the aromatic side chains) can, in turn, form β-sheet-rich filaments leading to the formation of nanotubes with a defined diameter (24 nm) and thickness and can subsequently lead to the formation of hydrogels. The structure of the semi-solid gel results from a very dense packing of the nanotubes [154,155]. The most important characteristic of the lancreotide-based hydrogel is that at low lanreotide concentration, the hydrogel slowly disassembles. This property is satisfactorily used to obtain a slow and controlled drug release that starts immediately upon transfer into a dilute solution. Depending on the lanreotide concentration, nanostructures in the semi-solid hydrogel can be arranged in a hexagonal lattice or form densely packaged structures (embedded nanotubes) [154].

Lanreotide was licensed by the FDA in 2007, and it is currently used for the relief of neuroendocrine symptoms and in particular for the treatment of radiotherapy-resistant acromegaly or in cases where surgery has failed to restore normal growth hormone secretion [156,157].

The trade name of the product is Somatuline^®^ Depot Injection. Somatuline Depot is a prescription medicine available as sterile and ready-to-use prefilled syringes containing lanreotide acetate supersaturated bulk solution of 24.6% *w*/*w* lanreotide base. It is safe and effective in adults, but, currently, it is not indicated in children [26,143,158].

## 6. Conclusions

Peptide self-assembly mechanisms represent a powerful tool to produce structures of varying size, scales, and functions and with exclusive physical properties. Protein structures are present in several biological systems as cargo transport, microbial defense, or structural support, ranging from oligomers and nanospheres to tubes or hierarchical assemblies. Usually, these structures are formed by non-covalent forces that give them dynamism and flexibility. Great attempts have been committed for exploring self-assembly proteins, which allow the formation of functional materials; however, the results are expensive and difficult to produce. In this context, short peptide-based structures have been shown to possess great potential as biomaterials representing a great promise for future research. The recent examples of successful clinical trials of the self-assembling peptide scaffolds for accelerating wound healing and regenerative medicine and surgical uses provide a glimpse of what is coming for their worldwide widespread use. In the future, SAP nanomaterials are required to be translated into commercial products providing, thanks to properties as specific as targetability, therapy, and eventual imaging ability, a functional improvement over existing products on the market. We believe that exploring new modifications in SAP sequences could be the key to creating novel structures able to be used in diverse fields.

## Figures and Tables

**Table 1:** Self-assembled peptides in the pharmaceutical market.

SAP	Sequence	Application	Aggregation Type	Reference
**RADA16**	RADARADARADARADA	Wound healing	Nanofibers	[24]
**P_11_-4**	QQRFEWEFEQQ	Dental caries	Nanotapes	[142]
**Lanreotide**	(D)βNal-CT-(D)W-KVCT	Neuroendocrine Tumors	Nanotubes	[143]

## Data Availability

Not applicable.
